# Engineering *Escherichia coli* towards *de novo* production of gatekeeper (2*S*)-flavanones: naringenin, pinocembrin, eriodictyol and homoeriodictyol

**DOI:** 10.1093/synbio/ysaa012

**Published:** 2020-08-06

**Authors:** Mark S Dunstan, Christopher J Robinson, Adrian J Jervis, Cunyu Yan, Pablo Carbonell, Katherine A Hollywood, Andrew Currin, Neil Swainston, Rosalind Le Feuvre, Jason Micklefield, Jean-Loup Faulon, Rainer Breitling, Nicholas Turner, Eriko Takano, Nigel S Scrutton

**Affiliations:** Manchester aaSynthetic Biology Research Centre for Fine and Speciality Chemicals (SYNBIOCHEM), Manchester Institute of Biotechnology and Department of Chemistry, The University of Manchester, Manchester M1 7DN, UK; Manchester aaSynthetic Biology Research Centre for Fine and Speciality Chemicals (SYNBIOCHEM), Manchester Institute of Biotechnology and Department of Chemistry, The University of Manchester, Manchester M1 7DN, UK; Manchester aaSynthetic Biology Research Centre for Fine and Speciality Chemicals (SYNBIOCHEM), Manchester Institute of Biotechnology and Department of Chemistry, The University of Manchester, Manchester M1 7DN, UK; Manchester aaSynthetic Biology Research Centre for Fine and Speciality Chemicals (SYNBIOCHEM), Manchester Institute of Biotechnology and Department of Chemistry, The University of Manchester, Manchester M1 7DN, UK; Manchester aaSynthetic Biology Research Centre for Fine and Speciality Chemicals (SYNBIOCHEM), Manchester Institute of Biotechnology and Department of Chemistry, The University of Manchester, Manchester M1 7DN, UK; Manchester aaSynthetic Biology Research Centre for Fine and Speciality Chemicals (SYNBIOCHEM), Manchester Institute of Biotechnology and Department of Chemistry, The University of Manchester, Manchester M1 7DN, UK; Manchester aaSynthetic Biology Research Centre for Fine and Speciality Chemicals (SYNBIOCHEM), Manchester Institute of Biotechnology and Department of Chemistry, The University of Manchester, Manchester M1 7DN, UK; Manchester aaSynthetic Biology Research Centre for Fine and Speciality Chemicals (SYNBIOCHEM), Manchester Institute of Biotechnology and Department of Chemistry, The University of Manchester, Manchester M1 7DN, UK; Manchester aaSynthetic Biology Research Centre for Fine and Speciality Chemicals (SYNBIOCHEM), Manchester Institute of Biotechnology and Department of Chemistry, The University of Manchester, Manchester M1 7DN, UK; Manchester aaSynthetic Biology Research Centre for Fine and Speciality Chemicals (SYNBIOCHEM), Manchester Institute of Biotechnology and Department of Chemistry, The University of Manchester, Manchester M1 7DN, UK; Manchester aaSynthetic Biology Research Centre for Fine and Speciality Chemicals (SYNBIOCHEM), Manchester Institute of Biotechnology and Department of Chemistry, The University of Manchester, Manchester M1 7DN, UK; MICALIS, INRA-AgroParisTech, Domaine de Vilvert, 78352 Jouy en Josas Cedex, France; Manchester aaSynthetic Biology Research Centre for Fine and Speciality Chemicals (SYNBIOCHEM), Manchester Institute of Biotechnology and Department of Chemistry, The University of Manchester, Manchester M1 7DN, UK; Manchester aaSynthetic Biology Research Centre for Fine and Speciality Chemicals (SYNBIOCHEM), Manchester Institute of Biotechnology and Department of Chemistry, The University of Manchester, Manchester M1 7DN, UK; Manchester aaSynthetic Biology Research Centre for Fine and Speciality Chemicals (SYNBIOCHEM), Manchester Institute of Biotechnology and Department of Chemistry, The University of Manchester, Manchester M1 7DN, UK; Manchester aaSynthetic Biology Research Centre for Fine and Speciality Chemicals (SYNBIOCHEM), Manchester Institute of Biotechnology and Department of Chemistry, The University of Manchester, Manchester M1 7DN, UK

**Keywords:** pathway engineering, flavonoids, synthetic biology, metabolic engineering, flavanones

## Abstract

Natural plant-based flavonoids have drawn significant attention as dietary supplements due to their potential health benefits, including anti-cancer, anti-oxidant and anti-asthmatic activities. Naringenin, pinocembrin, eriodictyol and homoeriodictyol are classified as (2*S*)-flavanones, an important sub-group of naturally occurring flavonoids, with wide-reaching applications in human health and nutrition. These four compounds occupy a central position as branch point intermediates towards a broad spectrum of naturally occurring flavonoids. Here, we report the development of *Escherichia coli* production chassis for each of these key gatekeeper flavonoids. Selection of key enzymes, genetic construct design and the optimization of process conditions resulted in the highest reported titers for naringenin (484 mg/l), improved production of pinocembrin (198 mg/l) and eriodictyol (55 mg/l from caffeic acid), and provided the first example of *in vivo* production of homoeriodictyol directly from glycerol (17 mg/l). This work provides a springboard for future production of diverse downstream natural and non-natural flavonoid targets.

## 1. Introduction

In nature, flavonoids are produced by a broad range of plant species to help combat various organic and non-organic stresses, including ultraviolet radiation, microbial infections, and both physical and radical damage. Humans consume flavonoids through plant-derived foods and these play crucial roles in helping prevent the onset of numerous diseases (1). Naringenin, pinocembrin, eriodictyol and homoeriodictyol are important naturally occurring flavonoids found in many edible fruits and plants, and they have far reaching applications in nutrition and human health. The beneficial effects of flavonoids have been well studied, and recent reviews have highlighted over 30 known applications for flavonoids in treating human disease, with properties ranging from anti-inflammatory, anti-hyperlipidemic, anti-cancer, anti-malarial and anti-stroke damage, to weight loss and radioprotection (DNA repair) (2–4). In addition, flavonoids are attracting research interest as potential biomaterials in tissue engineering applications (5). Current industry production of flavonoids comes from extraction from plant-based sources or *de novo* chemical synthesis; however, both routes have their disadvantages. The limited availability of flavonoids in plant tissues, prolonged and unpredictable crop harvesting and multiple solvent-based purification steps affect the yield and cost of production (6). In contrast, chemical synthesis requires the use of toxic solvents and extreme chemical reaction conditions, which is unsustainable and yields a non-green product, which can limit downstream applications (7). Biosynthesis via microbial fermentation offers an attractive alternative, with great strides having been made over the last 10 years in engineering biological systems to produce a number of industrial targets. Engineering microbial hosts for the production of fine and specialty chemicals, including flavonoids, offers an attractive and green route to these compounds and has become an ongoing focus of the synthetic biology community (8, 9).

**Fig 1:**
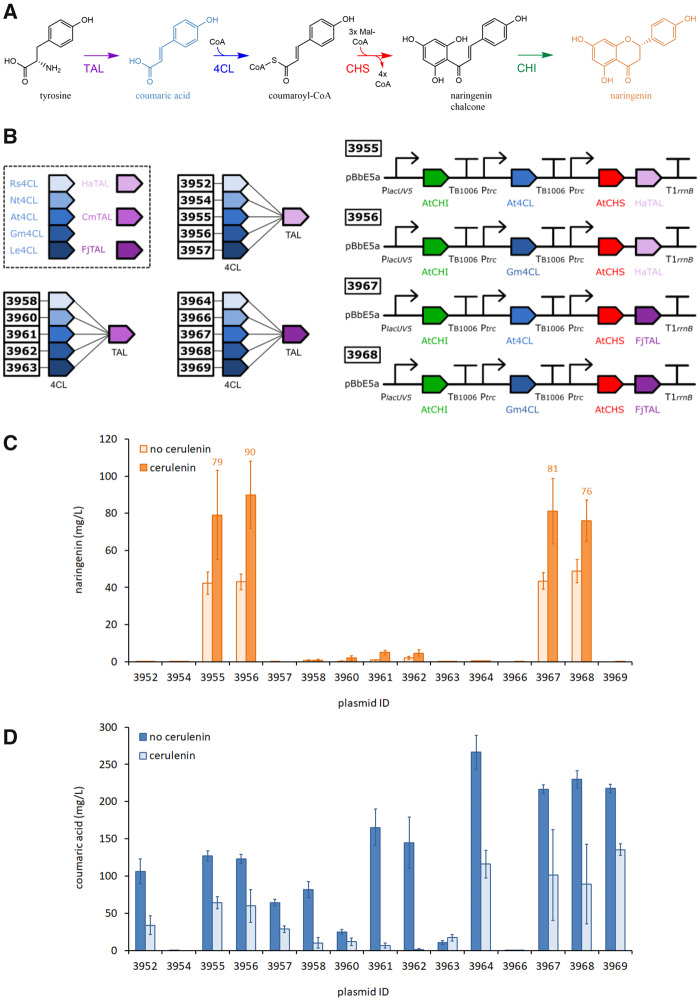
Enzyme selection and naringenin pathway construction. (**A**) Pathway highlighting key enzyme steps from tyrosine to naringenin. Enzyme abbreviations: TAL, tyrosine ammonia-lyase; 4CL, 4-coumarate-CoA-ligase; CHS, chalcone synthase; CHI, chalcone isomerase. (**B**) Construction of 15 Naringenin pathways with their corresponding plasmid IDs. Enzyme abbreviations: Rs4CL, 4CL from *R. sphaeroides*; Nt4CL, *N. tabacum*; At4CL, *A. thaliana*; Gm4CL, *G. max*; Le4CL, *L. erythrorhizon*; HaTAL, TAL from *H. aurantiacus*; CmTAL, *C. metallidurans*; FjTAL, *F. johnsoniae*. The four best-performing plasmid constructs are shown. (**C**) Screening a library of 15 pathways for the production of naringenin in the presence or absence of cerulenin. (**D**) Coumaric acid titers quantified for the same constructs. Data represent the mean and standard deviation from four replicate cultures (wildtype MG1655 cells grown at 30°C for 24 h in LB media with 0.4% glycerol and 3 mM tyrosine).

Four major flavonoids, naringenin, pinocembrin, eriodictyol and homoerydictiol, act as gatekeeper molecules due to their pivotal positions at important branch points in plant flavonoid biosynthesis pathways. Naringenin is commercially produced by extraction from grapefruit peel (*Citrus* × *paradisi* L.) a waste product of the juicing process (10), pinocembrin is obtained from *Populus* and *Euphorbia* plants (11), and both eriodictyol and homoeriodictyol are flavanones primarily extracted from the mountain balm plant (*Eriodictyon californicum*) or produced by multi-step chemical synthesis (12). Homoeriodictyol has many health-promoting properties including anti-oxidation, anti-inflammatory, anti-bacterial and anti-cancer effects (2, 3, 13). In addition, homoeriodictyol has applications as a taste enhancer/modifier for the food industry, and has the useful property of masking the bitter taste of foods without imparting any strong flavor of its own (14). In plants, flavanones are naturally produced from L-tyrosine (naringenin, eriodictyol, homoeriodictyol) or L-phenylalanine (pinocembrin), through the action of tyrosine ammonia-lyase (TAL) or phenylalanine ammonia-lyase (PAL), 4-coumarate-CoA ligase (4CL), chalcone synthase (CHS) and chalcone isomerase (CHI) (Figure 1A).

Bioproduction routes to flavonoids production have also been reported using *trans*-phenylacrylic acid precursors, such as coumaric acid, cinnamic acid, caffeic acid and ferulic acid, through metabolically engineered strains of *Escherichia coli*. Naringenin titers of 474 mg/l have been reported from coumaric acid (2.6 mM) (15). Feeding cinnamic acid has resulted in pinocembrin titers of 429 mg/l (16). Homoeriodictyol, methylated at the 3′ position, has been produced from eriodictyol by bioconversion using 3′-OMT from rice or tomatoes (17), and more recently from ferulic acid producing 52 mg/l homoeriodictyol (18). Bioproduction of eriodyctiol from caffeic acid has been previously reported (11 mg/l), and through extensive manipulation of malonyl-CoA biosynthesis combined with fine-tuning of *E. coli* metabolic pathways this has been improved to 50 mg/l (19) and 107 mg/l (20). Bioproduction of flavonoids directly from central metabolism has proven more challenging. Studies describing naringenin and pinocembrin production have reported titers of 100 mg/l (21) and 97 mg/l (22), respectively, requiring *matB* (malonyl-CoA synthetase) and *matC* (malonate carrier protein) genes, or genes encoding the subunits of acetyl-CoA carboxylase (*accA*, *accB*, *accC*, and *accD*) to boost malonyl-CoA availability.

Here, we demonstrate *E. coli* production chassis and optimized processes towards the overproduction of four key gatekeeper flavonoids. Utilizing a semi-automated Design-Build-Test-Learn (DBTL) synthetic biology pipeline, we present microbial production strains towards the *de novo* production of naringenin, pinocembrin, eriodictyol and homoeriodictyol, removing the need for precursor feeding. We report competitive production titers for naringenin and pinocembrin, directly from glycerol with titers of 484 mg/l and 214 mg/l, respectively. We demonstrate production of eriodictyol from caffeic acid (88 mg/l). And finally, we show the first example of homoeriodictyol production (17 mg/l) from central metabolism using glycerol as a carbon source, overcoming the need for *trans*-phenylacrylic acid feedstocks, by coupling to a 3-step ferulic acid production pathway. This study provides a platform for further downstream modification by enzymes to support the production of natural and non-natural flavanones, flavones, flavonols and isoflavones.

## 2. Materials and methods

### 2.1 Bacterial strains and media

*Escherichia coli* DH5α (New England Biolabs) was used for routine cloning and pathway propagation. Strains were maintained on Lysogeny broth (LB) or LB agar containing antibiotics for plasmid selection. Production experiments were conducted in a variety of media and included: Terrific broth (TB, Formedium TRB0102); TBP (TB phosphate-buffered, Formedium TBP0102); TBsb (TB supplemented with 0.5 M sorbitol and 5 mM betaine), EZ (EZ-rich defined medium kit, Teknova M2105), MOPS (prepared as EZ but excluding 10× ACGU and 5× Supplement EZ), Super Optimal Broth (SOB, Formedium SOB0202) and M9 (23). All media were supplemented with either 0.4% w/v glucose or glycerol, and antibiotics as appropriate: ampicillin (100 µg/ml), kanamycin (50 µg/ml) and/or chloramphenicol (20 µg/ml).

### 2.2 Plasmid assembly

The pathways were built using an automated ligase cycling reaction assembly method as described previously (8, 9, 24). Automated, worklist-driven liquid handling was implemented for bridging oligo pooling and ligase cycling reaction setup. Completed reactions were transformed into high-efficiency NEB 5-alpha cells, and correct plasmid assemblies identified by high-throughput, next-generation sequencing using Nanopore technology (18). Some assemblies (Supplementary Table S3) were also verified using Sanger sequencing (GATC Biotech). Through this assembly pipeline, we typically obtained >70% of sequence-perfect plasmid targets at the first attempt. Routine sub-cloning of pathways into different plasmid vectors, and replacing genes (4CL and CHS gene libraries) in pre-existing plasmid pathways, was performed using an In-Fusion HD Cloning kit (Takara Bio Inc.).

### 2.3 *In vivo* production experiments

Overnight seed cultures were grown from freshly transformed colonies in the desired production media at 37°C with shaking at 180 rpm. To help maintain a consistent biomass, seed cultures were normalized to OD_600_ = 1.0 before inoculating 1/50 into fresh media (1.2 ml in 96-deepwell blocks with breathable seals) and grown at 30°C, 950 rpm shaking, 75% humidity. When cultures reached OD_600_ = 1.5–2.0 (or OD_600_ = 0.5–0.6 for MOPS and M9 minimal media) they were induced with the addition of 100 µM IPTG, 3 mM substrate (tyrosine, phenylalanine or *trans*-phenylacrylic acids) and 20 µg/ml cerulenin, as appropriate, then transferred back to the 30°C shaker-incubator for 24 h. Cultures were processed for mass spectrometry analysis as described below and, if necessary placed back in the 30°C shaker-incubator for further processing at later timepoints. All liquid handling steps for plate-based experiments were performed on a Hamilton Star robotic platform.

### 2.4 Analysis and quantification of target compounds

For all flavonoid pathways involved in this study, the *trans*-phenylacrylic acid intermediates and corresponding targeted flavanones were quantified. A comprehensive MS method covering all potential products was developed. For consistency and sample throughput, a Hamilton robotic platform was used to process all MS samples. Briefly, cell cultures were quenched with an equal volume of 100% methanol. Samples were then centrifuged at 2250 RCF for 10 min (5804 R centrifuge; Eppendorf, Germany). Supernatants were extracted and diluted as required with methanol/water (10:90 v/v) in 96-well plates. These plate-based extractions were designed to be compatible for analysis on a triple quadrupole tandem mass spectrometer (Xevo TQ-S; Waters MS Technologies) connected to an Acquity Ultra Performance Liquid Chromatography system (Acquity UPLC; H-Class, Waters) with a 96-well plate sample tray accessory. UPLC methods were optimized on a BEH C18 column (2.1 × 50 mm, 1.7 μm; Waters) for the resolution of *trans*-phenylacrylic acid intermediates: cinnamic acid, coumaric acid, caffeic acid, ferulic acid, 4-methoxycinnamic acid; and final flavanones pinocembrin, naringenin, eridictyol, homoeridictyol and isosakuranetinon. Mobile phase A (water + 0.05% formic acid) and mobile phase B (methanol + 0.05% formic acid) were used at an operating temperature of 45°C. Instrument injection sequences were randomly generated by in-house Excel-based data tracking and worklist generators. The optimized flavonoids LC gradient ran at a flow rate of 0.6 ml min^−1^, resulting in a total runtime of 2 min per sample. A linear gradient of 40–95% B (v/v) was applied over 1.5 min, before returning linearly to 40% B (v/v) over 1 min. A total of 40% B (v/v) was then maintained for 0.4 min to equilibrate the system for the next injection. The MS parameters were optimized with a desolvation gas flow of 1000 l h^−1^, a capillary voltage of 1 kV, desolvation temperature of 600°C and source temperature set to 150°C. The quantification MRM transitions for all intermediates and final products were resolved with standard curves. The key parameters such as precursor ion, daughter ion, cone voltage and collision energy (CE) are shown in Supplementary Table S5. The limit of detection (LOD) and limit of quantification (LOQ) were calculated from the signal-to-noise ratio (S/N), the LOD was defined when S/N > 3 and the LOQ defined when S/N > 10, which were used to generate the workable linear range of the calibration curve for quantifying intermediates and flavonoids. An external 8-point calibration curve was used to analyze the whole library. All standard stock solution were freshly prepared in ethanol, then diluted to test the concentration range with 0.2% TB media in MeOH/H_2_O (10:90 v/v) and with pure MeOH/H_2_O (10:90 v/v) to assess matrix effects from the culture medium. No significant matrix effects were observed with the dilution factors used in this study (16 000×–40 000×). Based on the LOQ of each compound with the developed method, a standard mixture was used to generate calibration standard curves for quantification, consisting of 5 µM cinnamic acid, 1 µM coumaric acid, 2 µM caffeic acid, 1 µM ferulic acid, 30 µM 4-methoxycinnamic acid, 200 nM pinocembrin, 100 nM naringenin, 200 nM eriodictyol, 200 nM homoeriodictyol and 200 nM isosakuranetin. The linear model for standard curves was selected based on the analysis of data by linear regression weighting factors 1/x, using MassLynx V4.1 SCN905, with TargetLynx (Waters Corp., Milford, MA, USA).

## 3. Results and discussion

### 3.1 Enzyme selection—Selenzyme

One key requirement in producing a well-balanced biosynthetic pathway is selecting optimal enzymes to catalyze each reaction step towards the production of the desired target compound. We previously constructed a pinocembrin production pathway and sought to modify this for naringenin production by exchanging two of the genes: to replace the PAL (EC 4.3.1.24) gene with different TAL genes (EC 4.3.1.23), and to test a range of different 4CL genes (EC 6.2.1.12)]. TAL catalyzes the conversion of tyrosine to coumaric acid, and the replacement of 4CL addresses a potential bottleneck during the conversion of 4-coumaric acid to coumaroyl-CoA. Databases of target enzymes were curated using Selenzyme (25) providing multiple scores for sequence selection based on several criteria, including reaction and sequence similarity, phylogenetic distance and known protein activity. These criteria were used to select a preliminary list of top enzyme candidates, based on reaction signatures, sequence similarity and kinetic information, followed by manual curation to select for known performance and phylogenetic diversity.

In the case of TAL, kinetic information was available for the three top candidates: HaTAL (from *Herpetosiphon aurantiacus*); CmTAL (*Cupriavidus metallidurans*) and FjTAL (*Flavobacterium johnsoniae*) (Supplementary Table S1). Therefore, their substrate specificity and enzyme efficiency were evaluated through comparison of the kinetic values for their ability to perform as either TALs, PALs or tyrosine ammonia mutases (TAMs), as shown in Supplementary Table S1. 4CL enzymes were selected by curation of the top candidates from Selenzyme, complimented with literature and database mining. The selection took into consideration the Selenzyme scores, compatibility, and substrate affinity. We selected five 4CL enzymes, including the top 4 ranked sequences: Rs4CL (from *Rhodobacter sphaeroides*), Nt4CL (*Nicotiana tabacum*), At4CL (*Arabidopsis thaliana*) and Gm4CL (*Glycine max*). We also included Le4CL (from *Lithospermum erythrorhizon*), due to its reported high promiscuity in accepting *trans*-phenylacrylic acid substrates for flavonoids and stilbenes production (26).

### 3.2 Pathway engineering

We based our naringenin pathway genetic architecture on our previously optimized pinocembrin production pathway (8). This pathway produced 88 mg/l of pinocembrin in an engineered *E. coli* strain supplemented with both phenylalanine (3 mM) and the fatty acid inhibitor cerulenin. To see if we could achieve similar success for naringenin, we switched the PAL and 4CL genes with the three TAL and five 4CL candidates to give a total of 15 pathway variants (Figure 1B). Due to the small library size, the full combinatorial library of expression constructs was assembled using our automated pathway assembly pipeline (8, 9, 27–29) (Supplementary Figure 1). In brief, gene parts were designed to be compatible with DNA assembly via the ligase cycling reaction, and *in silico* generated pipetting worklists were generated to drive the liquid handling robotic platforms. Successful assembly of pathway plasmids was confirmed by multiplexed sequence verification by NGS methods.

The 15 pathways were screened in *E. coli* DH5α for *in vivo* production of naringenin during fermentation in liquid cultures supplemented with 3 mM tyrosine and 20 μg/ml cerulenin (Figure 1C and D). Of the 15 pathways, four produced significant amounts of naringenin: plasmids 3955 (79 mg/l), 3956 (90 mg/l), 3967 (81 mg/l) and 3968 (76 mg/l). These pathways all contained either FjTAL or HaTAL, in combination with either Gm4CL or At4CL. Pathways that contained either Rs4CL, Nt4CL or Le4CL failed to produce significant amounts of naringenin. Surprisingly, Rs4CL which scored highest in our *in silico* predictions, was one of the poorest performers *in vivo* (0.1–4.5 mg/l in plasmids 3952, 3958 and 3964). Gm4CL had the lowest *K*m for coumaric acid (0.017 µM) and proved to be the best-performing 4CL tested (plasmids 3956 and 3968). Constructs containing CmTAL showed low levels of naringenin production (<5 mg/l), even when paired with Gm4CL or At4CL, perhaps due to the significantly lower catalytic efficiency of CmTAL (*K*cat/*K*m 0.031 mM^−1^ s^−1^) compared to FjTAL (*K*cat/*K*m 2.99 mM^−1^ s^−1^) and HaTAL (*K*cat/*K*m 2.74 mM^−1^ s^−1^) (Supplementary Table S1).

Having assembled and tested these initial pathways for naringenin production, we anticipated that significant enhancements in target titers could be achieved through selected optimization of process conditions and genetic construct design. For example, through: (i) selection and engineering of host strains to improve metabolic flux and substrate availability; (ii) fine-tuning the fermentation process conditions and (iii) improvements in the genetic construct design. To test this, we chose our best four performing pathways (plasmids 3955, 3956, 3967 and 3968) for further downstream optimization.

### *3.3 Escherichia coli* strain selection

**Fig 2:**
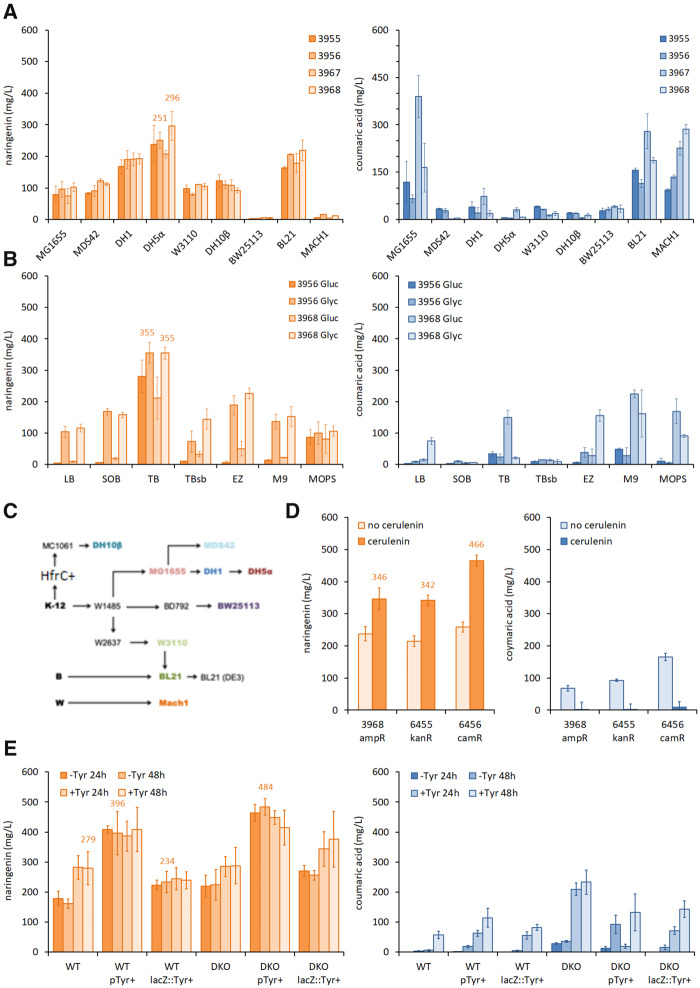
Optimization of naringenin pathway constructs and process development. (**A**) Screening of naringenin pathway constructs in nine common *E. coli* strains. Naringenin and coumaric acid titers were quantified for cultures grown in the presence of cerulenin. (**B**) Media and carbon source screening. Naringenin pathway constructs in DH5α were screened for naringenin and coumaric acid production in seven different culture media with 0.4% w/v of glucose or glycerol, in the presence of cerulenin. (**C**) Pedigree chart for the *E.*  *coli* strains screened for narigenin production. (**D**) Screening a naringenin pathway in vectors with different antibiotic-resistance genes. Naringenin and coumaric acid titers were quantified for cultures grown in the presence or absence of cerulenin. (**E**) The best preforming naringenin construct (6456) screened for naringenin and coumaric acid production in DH5α wildtype (WT) and double knockout (DKO; Δ*tyrR*, Δ*pheLA*) strains, with or without a tyrosine overproduction construct as free plasmid (pTyr+) or genome integrated (*lacZ::*Tyr+). Data represent the mean and standard deviation from four replicate cultures (wildtype DH5α cells grown at 30°C for 24 h in TBP media with 0.4% glycerol and 3 mM tyrosine, unless otherwise indicated).

First, we tested our top pathways in 9 common laboratory *E. coli* strains using the same initial production conditions. This included the K-12 strains MG1655, MDS42, DH1, DH5α, W3110, DH10B and BW25113; the B strain BL21; and the W strain Mach1. We anticipated only subtle differences in target production due to the high genome sequence similarity and close genetic lineage of these lab-adapted *E. coli* strains (Figure 2C). All K-12 strains, with the exception of BW25113, showed good production of naringenin: DH5α was the best-performing chassis tested, producing 296 mg/l (plasmid 3968) and 251 mg/l (plasmid 3956) of naringenin, an almost 4-fold increase compared to our initial screening strain MG1655. DH1 and BL21 showed similar titers across the four pathways, an approximately 2-fold increase compared to MG1655. Production strains were ranked as follows: DH5α; DH1; BL21; MG1655; MDS42; W3110; DH10B; Mach1; BW25113 (Figure 2A). It is noteworthy that coumaric acid did accumulate for some of the strains, suggesting that the pathway bottleneck lies further downstream of this intermediate, perhaps through malonyl-CoA availability.

We next tested our best producing strain (DH5α) in seven commonly available culture media, using both glycerol and glucose as a carbon source. The pathway and strain selection screens above were done in TB media, and this media proved optimal for naringenin production under our fermentation conditions. Glucose proved to be a poorer carbon source than glycerol in all media types. TB media with 0.4% w/v glycerol gave the highest naringenin titer of 355 mg/l (for pathways 3956 and 3968). Media conditions were ranked as follows: TB; Ez-rich; SOB; TBsb; M9; MOPS; LB and carbon source glycerol > glucose (Figure 2B).

### 3.4 The impact of antibiotic-resistance marker

We next looked at additional improvements to our genetic construct design. Protein synthesis rates have been shown to be sensitive to the use of different antibiotic-resistance markers (1.5-fold variation) (30), therefore, we chose to test three different resistance markers for their effect on naringenin production. The most productive naringenin pathway 3968, in the pBbE5a vector (ampicillin resistance), was subcloned into pBbE5k (6455; kanamycin) and pBbE5c (6456; chloramphenicol). All plasmids were screened in DH5α, for naringenin and coumaric acid production (Figure 2D) and assessed on their production efficiency at varies induction ODs (Supplementary Figure 8). It is noteworthy, that higher starting biomass (2–4 OD_600_) had a negative effect on naringenin production, although a significant increase of coumaric acid was observed at these higher induction points. Moreover, the catalytic activity of CHI is pH-dependent. For example, the activity at pH 6 is roughly 50% of that at pH 7 (31). This may help explain, at least in part, the need to regulate pH levels during fermentation runs—a strategy that has seen significant increases in flavonoid production (32). Antibiotic-resistance cassette preference followed the order *cam^R^* > *kan^R^* > *amp^R^*. The best-performing plasmid pathway pBbE5c (6456), carrying the FjTAL and Gm4CL genes, produced 466 mg/l naringenin and so was taken forward into the next phase of strain optimization.

### 3.5 Chassis engineering towards tyrosine overproduction

Having established the optimal genetic pathway construct and best fermentation conditions, we looked to streamline our fermentation conditions by removing additional feedstock requirements. We previously constructed a gene double knockout (DKO) strain to boost the metabolic flux towards tyrosine overproduction (Δ*tyrR*, Δ*pheLA*) (9). We deleted the *tyrR* gene, a transcriptional regulator of aromatic amino acid biosynthesis, from the DH5α genome and then deleted *pheLA* (bifunctional chorismate mutase/prephenate dehydratase) to boost tyrosine levels and restrict phenylalanine production (Supplementary Figure S7). We also used an additional expression plasmid (5753, pTyr+) carrying the E. coli ppsA, aroG* and tyrA* genes (* feedback-resistant mutants), to help focus metabolic flux towards tyrosine for our naringenin pathways. The pTyr+ construct was also integrated into the *lacZ* locus in the chromosome of wildtype (WT) and DKO mutant DH5α cells. We initially compared the efficiency of genome integration versus plasmid-borne delivery of the pTyr+ construct in both WT DH5α and DKO strains carrying the naringenin pathway (plasmid 6456) (Fig 2E). Naringenin and coumaric acid titers were determined at 24 h and 48 h time points, but no significant accumulation of these targets was observed after 24 h. Genome integration of the tyrosine construct (*lacZ*::Tyr+) in WT DH5α supported a modest 234 mg/l of naringenin, compared to 396 mg/l when delivered by plasmid (pTyr+) in the WT strain (Figure 2E). In this case, the single genomic copy of pTyr+ appeared unable to fully compensate for the absence of tyrosine supplementation (*cf*. 279 mg/l naringenin for WT DH5α with 3 mM tyrosine). In future, this may be overcome by increasing the promoter strength of the genome-integrated construct. Inclusion of pTyr+ increased the titers to 409 mg/l in WT DH5α, but in combination with the DKO strain produced 484 mg/l naringenin, our highest titer in this study. It is noteworthy that coumaric acid concentrations were relatively low (<40 mg/l) for all strains that were not supplemented with tyrosine, suggesting that tyrosine availability may still be a limiting factor that could be addressed by further strain engineering (Figure 2E). To our knowledge, pTyr in combination with the DKO produced the highest titer reported for naringenin production from primary metabolism in *E. coli*. Previously, Wu *et al.* showed production of 101 mg/l naringenin directly from glucose (21), and prior highest titers of 421 mg/l were reported with tyrosine addition (3 mM) (33), and 474 mg/l from coumaric acid feeding (2.6 mM) (15). Recently *Palmer et al.* reported shake-flask production of 124 mg/l of naringenin from tyrosine in the yeast strain *Yarrowia lipolytica*. Applying this strain in a batch-fed 3 l bioreactor, increased production to an impressive 898 mg/l. However, this required the addition of 220 g/l D-glucose over a 12 day period (34).

### 3.6 Optimization of pinocembrin pathways

**Fig 3:**
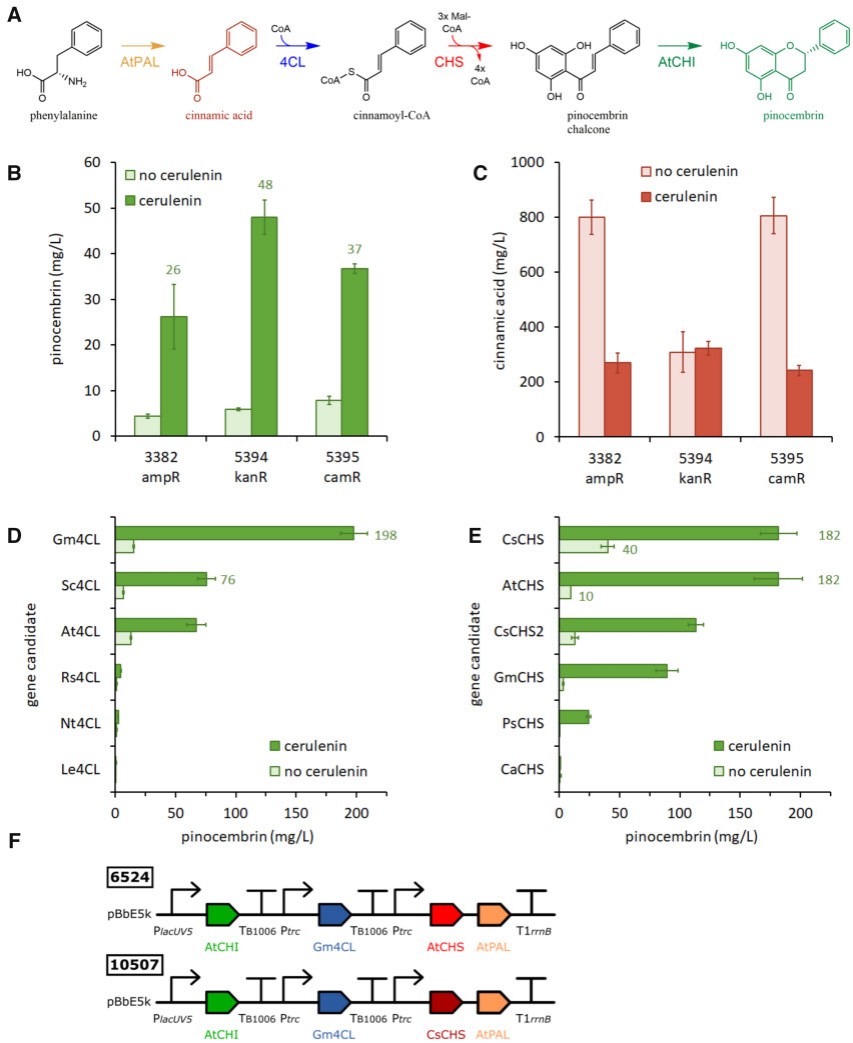
Optimization of pinocembrin pathway constructs. (**A**) Pathway from phenylalanine to pinocembrin. Enzyme abbreviations: AtPAL (phenylalanine ammonia-lyase from *A. thaliana*); 4CL (4-coumarate-CoA-ligase); CHS (chalcone synthase); AtCHI (chalcone isomerase from *A. thaliana*). (**B**) Screening a pinocembrin pathway in vectors with different antibiotic-resistance genes. Pinocembrin titers were quantified for cultures grown in the presence or absence of cerulenin. (**C**) Cinnamic acid titers quantified from the same cultures. (**D**) Screening pinocembrin pathways with different 4CL gene candidates in the presence or absence of cerulenin. (**E**) Screening pinocembrin pathways with different CHS gene candidates in the presence or absence of cerulenin. (**F**) Best-performing pinocembrin constructs. Data represent the mean and standard deviation from four replicate cultures (wildtype MG1655 cells grown at 30°C for 24 h in TBP media with 0.4% glycerol and 3 mM phenylalanine).

Previously, we have used our automated DBTL pipeline to construct *E. coli* strains that produce pinocembrin (8). We optimized the gene/promoter structure of the 4-gene pathway (Figure 3A), identified the best replication origin, and screened an *E. coli* strain library to identify the most suitable host (MG1655). Our best pathway produced 29 mg/l of pinocembrin in WT MG1655 grown in TBP media supplied with 0.4% glycerol and 3 mM phenylalanine. In light of the far higher naringenin titers described above, we re-evaluated our pinocembrin pathways to see whether we could achieve similar titers. One variable not tested previously was the effect of the plasmid antibiotic-resistance marker (30). Therefore, we subcloned our best pathway from its original pBbE5a vector (3382, *amp^R^*) into pBbE5k (5394, *kan^R^*) and pBbE5c (5395, *cam^R^*), to test how antibiotic selection affects final titers. The three plasmids were screened for production of pinocembrin (Figure 3B) and cinnamic acid (Figure 3C), in MG1655 cells grown in TBP media with 0.4% glycerol and 3 mM phenylalanine. The vector containing the kanamycin resistance marker was the most productive plasmid (48 mg/l pinocembrin after 24 h), followed by *cam^R^* (37 mg/l) and *amp^R^* (26 mg/l). Pinocembrin titers were 4- to 8-fold higher in the presence of the fatty acid biosynthesis inhibitor cerulenin. Notably, all three plasmids accumulated significant amounts of the intermediate cinnamic acid (240–800 mg/l), which is known to adversely affect pinocembrin production (32).

Cinnamic acid is produced by PAL (AtPAL from *A. thaliana*) and converted to cinnamoyl-CoA by 4CL (Sc4CL from *Streptomyces coelicolor* in the plasmids above). We investigated whether Sc4CL was a limiting step in our pinocembrin pathways by screening a panel of six alternative 4CL candidates for their ability to support pinocembrin production. The 4CL genes were each cloned into the pinocembrin pathway on the *kan^R^* vector (5394), to replace the Sc4CL gene in this construct. Plasmids were screened in MG1655 as above (Figure 3D), with only one (6524, Gm4CL) producing more pinocembrin than the parent plasmid (5394, Sc4CL). Gm4CL from *Glycine max* (soybean) yielded a 2.6-fold higher pinocembrin titer than Sc4CL in the same pathway (198 mg/l versus 76 mg/l) and was also the best 4CL homologue identified from screening the naringenin plasmid library (Figure 1C). Given this significant increase in pinocembrin production, we next chose to screen a panel of six alternative CHS candidates (selected using Selenzyme) to see whether this was now the limiting step in our pathway. The CHS genes were each cloned in the Gm4CL-containing pathway construct (6524) and again screened in MG1655 (Figure 3E). Amongst this set of plasmids, only one (10 507, CsCHS) performed as well as the parent plasmid (6524, AtCHS), producing 182 mg/l pinocembrin in the presence of cerulenin. However, in the absence of cerulenin the CsCHS-containing plasmid produced a 4-fold higher pinocembrin titer than the AtCHS equivalent (40 versus 10 mg/l). The superior function of CsCHS from *Camellia sinensis* (Tea) in the absence of cerulenin suggests that this enzyme competes more effectively with fatty acid biosynthesis for available malonyl-CoA. We further tested the library of variant CHS pathways for the production of naringenin, by providing 3 mM coumaric acid in the culture media to bypass the ammonia-lyase step (Supplementary Figure S2). In the absence of cerulenin, the CsCHS pathway produced 179 mg/l naringenin, compared to 88 mg/l for the AtCHS pathway. Cerulenin is an expensive additive to fermentation media, so pathways employing CsCHS should be useful in future strategies to improve the economics of flavonoid production.

### 3.7 Other (2S)-flavanone targets

**Fig 4:**
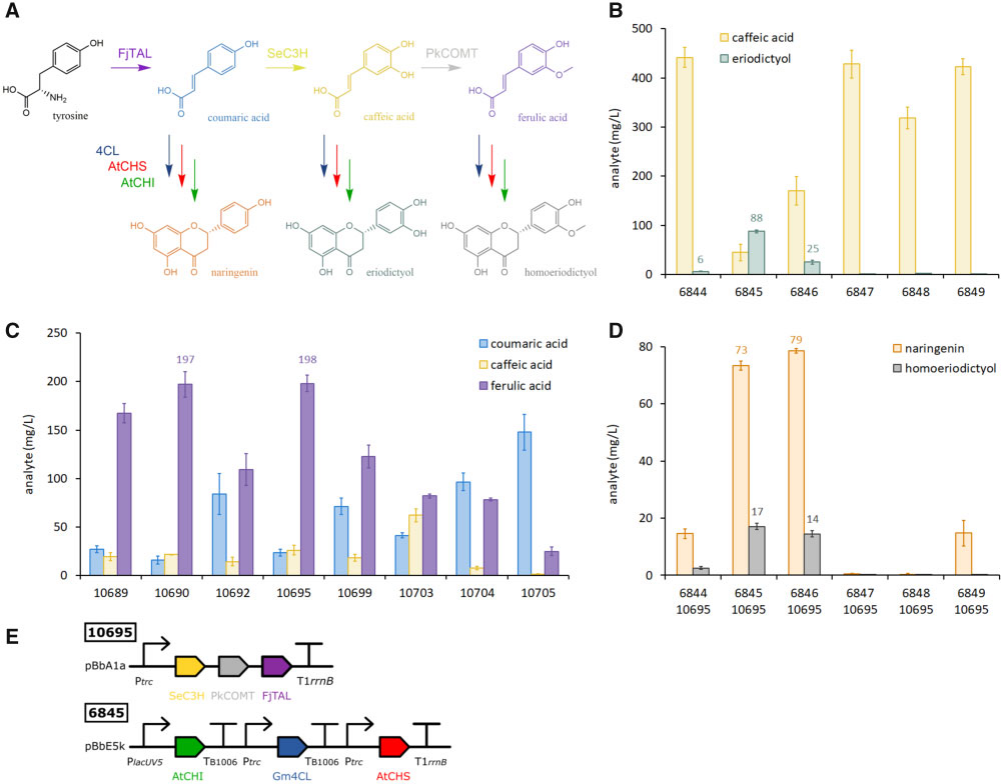
*Escherichia coli* production of eriodictyol and homoeriodictyol. (**A**) Pathways from tyrosine to the (2*S*)-flavanone targets naringenin, eriodictyol and homoeriodictyol. New enzyme abbreviations: SeC3H (coumarate 3-hydroxylase from *S. espanaensis*); PkCOMT (caffeate 3-*O*-methyltransferase from *P. kitakamiensis*). (**B**) Screening a panel of pathways with different 4CL gene candidates for production of eriodictyol from 3 mM caffeic acid substrate, in the presence of cerulenin. (**C**) Screening a library of ferulic acid pathway constructs for the production of *trans-*phenylacrylic acids from 3 mM tyrosine substrate. (**D**) Screening a library of (2*S*)-flavanone pathway constructs (different 4CL genes) with the best-performing ferulic acid pathway, for production of homoeriodictyol and naringenin (in the presence of cerulenin but without tyrosine). (**E**) Best-performing constructs for production of homoeriodictyol. Data represent the mean and standard deviation from four replicate cultures (wildtype DH5α cells grown at 30°C for 24 h in TBP media with 0.4% glycerol).

The pathways which produce naringenin (6456) and pinocembrin (6524) differ only in the ammonia-lyase gene expressed, FjTAL or AtPAL, respectively. The specificity of these two enzymes is very tight, even when supplied with substrate, naringenin pathways do not produce pinocembrin from phenylalanine and pinocembrin pathways do not produce naringenin from tyrosine (<1 mg/l; Supplementary Figure S3). The remaining three enzymes (Gm4CL, AtCHS, AtCHI) are common to both pathways, converting *trans*-phenylacrylic acid intermediates into their respective (2*S*)-flavanones. To determine whether these three enzymes can process other *trans*-phenylacrylic acid substrates, we deleted the PAL gene from the 6 pinocembrin plasmids that were used to screen 4CL gene candidates (Figure 3D). We transformed MG1655 cells with these ΔPAL constructs and supplemented media with 3 mM of various *trans*-phenylacrylic acids, screening for substrate depletion and accumulation of the expected (2*S*)-flavanones (Supplementary Figure S4). As expected, cinnamic acid was converted to pinocembrin (214 mg/l) and coumaric acid to naringenin (199 mg/l), with Gm4CL being the most productive 4CL variant for both targets. This Gm4CL plasmid (6845) was also the most efficient at converting caffeic acid to eriodictyol (88 mg/l; Figure 4B); however, this represents just 18% of the expected yield from the amount of caffeic acid consumed. Eriodictyol and caffeic acid both contain catechol moieties which are readily oxidized to *ortho*-quinones (35) that react with cysteine residues of proteins, the formation of these reactive species would reduce the amount of detectable eriodictyol in our media. Future optimization of eriodictyol production will require strategies to prevent oxidation of this target during fermentation. Eriodictyol has been produced in *E. coli* at 110 mg/l from tyrosine, via P450-mediated hydroxylation of naringenin in a strain engineered to enhance availability of malonyl-CoA (20). Our titer of 88 mg/l eriodictyol in WT cells suggests that alternative routes through caffeic acid, thus avoiding problematic P450 enzymes, might be more amenable to optimization. The ΔPAL constructs were also screened for the conversion of ferulic acid to homoeriodictyol, and 4-methoxycinnamic acid to isosakuranetin, but neither of these methylated flavanones was produced at mg/l scale (Supplementary Figure S4). Ferulic acid was depleted by cells carrying the Gm4CL (6845) and At4CL (6846) plasmids, but only 0.5 mg/l of homoeriodictyol was detected. In contrast, 4-methoxycinnamic acid was not consumed by any of the ΔPAL pathways, suggesting that the 4CL candidates tested cannot accommodate this substrate.

### *3.8 Escherichia coli* production of homoeriodictyol

Although we only produced 0.5 mg/l homoeriodictyol from our ΔPAL pathways, Cui *et al.* (18) recently reported the production of 52 mg/l homoeriodictyol by feeding ferulic acid at lower concentrations. Therefore, we investigated the construction of pathways to allow the complete biosynthesis of homoeriodictyol from tyrosine, with the rationale that ferulic acid produced *in situ* would be quickly converted on to homoeriodictyol, preventing build-up that may inhibit pathway function. First, we designed a 12-member library of ferulic acid pathway plasmids (Supplementary Figure S5), combining FjTAL with coumarate 3-hydroxylase (SeC3H) from *Saccharothrix espanaensis* (36), and caffeate 3-*O*-methyltransferase (PkCOMT) from *Populus kitakamiensis* (9). These pathways should catalyze the sequential conversion of tyrosine to coumaric acid, caffeic acid and finally ferulic acid (Figure 4A). We successfully assembled eight sequence-verified plasmids from the 12-member library and screened DH5α transformants for the production of ferulic acid from tyrosine (Figure 4C). All eight pathways produced ferulic acid (25–198 mg/l), with plasmids 10 690 and 10 695 providing the highest titers. There were also significant amounts of coumaric acid and caffeic acid in the media after the 24 h culture period, indicating that conversion was not complete. However, plasmids 10695 (198 mg/l ferulic acid vs. 49 mg/l coumaric plus caffeic acid) and 10 690 (197 mg/l vs. 37 mg/l) had the best conversion efficiency. Ferulic acid has previously been produced at 196 mg/l in shake-flask cultures of an *E. coli* tyrosine overproducer strain (37), and so our equivalent titers in DH5α compare favorably.

To express the full homoeriodictyol pathway in *E. coli*, we co-transformed DH5α cells with the 3-gene ferulic acid plasmid (10 695) and each of the 3-gene ΔPAL plasmids (6844–6849). Homoeriodictyol was produced at the highest levels by pathways including the Gm4CL (17 mg/l) and At4CL (14 mg/l) genes (Figure 4D). These titers were for WT DH5α cells supplied with 0.4% glycerol in the presence of cerulenin. To our knowledge, this is the first report of the complete biosynthesis of homoeriodictyol in *E. coli*. Naringenin was also detectable in the culture media (73–79 mg/l), this was expected because coumaric acid is a precursor to ferulic acid, and the 4CL, AtCHS and AtCHI enzymes can process both compounds (Figure 4A). Presumably eriodictyol was also produced, although we did not monitor this side-product. We investigated whether supplementing culture media with 3 mM tyrosine would boost final titers of homoeriodictyol, but these were actually slightly reduced (11–12 mg/l), whilst more naringenin was produced (105–123 mg/l; Supplementary Figure S6). Future improvements to *E. coli* production of homoeriodictyol will require further screening or engineering of 4CL candidates, to identify enzymes that effectively discriminate between coumaric acid, caffeic acid and ferulic acid. The homoeriodictyol pathway we report here could also be adapted for production of eriodictyol, through deletion of the PkCOMT gene in these constructs, combined with strategies to prevent oxidation of this labile target.

In conclusion, we demonstrate *E. coli* production chassis and optimized processes towards the overproduction of 4 key gatekeeper flavonoids. We have assembled and screened biosynthetic pathway constructs towards the *de novo* production of naringenin, pinocembrin, eriodictyol and homoeriodictyol in *E. coli* host strains, removing the need for precursor feeding for 3 out of the 4 targets. These strains were able to successfully produce naringenin and pinocembrin with titers reaching 484 mg/l and 198 mg/l, respectively. We demonstrate the production of eriodictyol (88 mg/l) from feeding caffeic acid. And finally, we reveal the first example of homoeriodictyol production (17 mg/l) from a simple carbon source (glycerol), overcoming the need for *trans*-phenylacrylic acid feedstocks by coupling to a ferulic acid production pathway. This resulting optimized production strains provide an ideal platform for further downstream expansion from these targets with additional enzymes to support the production of natural and non-natural flavonoids, such as tailored flavanones (methylation, glycosylation, chlorination and prenylation) or alternative flavonoid scaffolds (flavones, flavonols and isoflavones).

## Orcid

Mark S. Dunstan: 0000-0002-9416-5505, Christopher J. Robinson 0000-0001-6146-566, Adrian J. Jervis 0000-0003-2386-3354, Cunyu Yan 0000-0002-3603-2421, Pablo Carbonell 0000-0002-0993-5625, Katherine A. Hollywood 0000-0002-7028-047X, Andrew Currin 0000-0001-7845-8837, Neil Swainston 0000-0001-7020-1236, Jason Micklefield 0000-0001-8951-4873, Jean-Loup Faulon 0000-0003-4274-2953, Rainer Breitling: 0000-0001-7173-0922, Nicholas J. Turner 0000-0002-8708-0781, Eriko Takano: 0000-0002-6791-3256, Nigel S. Scrutton: 0000-0002-4182-3500.

## Funding 

This work was supported by the Biotechnology and Biological Sciences Research Council (BBSRC) and the Engineering and Physical Sciences Research Council (EPSRC) under grants: ‘Centre for synthetic biology of fine and specialty chemicals (SYNBIOCHEM)’ (BB/M017702/1) and ‘Future Biomanufacturing Research Hub’ (EP/S01778X/1). This project also received funding from the European Union’s Horizon 2020 research and innovation programme under grant agreement number 814408 (ShikiFactory100). J.L.F acknowledges funding provided by the French National Funding Agency under grant agreement number ANR-15-CE1-0008.

*Conflict of interest statement*. None declared.

## Supplementary Material

ysaa012_Supplementary_Data
