# Supplementary material for: Engineering Escherichia coli towards de novo production of gatekeeper (2S)-flavanones: naringenin, pinocembrin, eriodictyol and homoeriodictyol
Source: Synth Biol (Oxf). 2020 Aug 6;5(1):ysaa012. doi: 10.1093/synbio/ysaa012 (PMC7644443; doi:10.1093/synbio/ysaa012)
Supplement: ysaa012_Supplementary_Data [file ysaa012_supplementary_data.zip › Dunstan et al supplemental data.docx]

**Sup Figure 1:** Illustration of the full combinatorial library of naringenin pathways. Fifteen pathways combining five 4CL and three TAL enzymes were built and tested. Plasmid numbers are given.

**Sup Figure 2**: Screening CHS genes for the production of naringenin. Pinocembrin pathways with different CHS gene candidates were supplied with 3 mM coumaric acid (these plasmids carry a PAL gene, rather than TAL, and so could not produce coumaric acid from tyrosine). Pathways were screened for the production of naringenin in the presence or absence of 20 µg/ml cerulenin. Data represent the mean and standard deviation from three replicate cultures (wildtype DH5α cells grown at 30 °C for 24 hr in TBP media with 0.4% glycerol).

**Sup Figure 3**: PAL/TAL specificity determines pathway production of pinocembrin or naringenin. All six plasmids carry the 4CL, CHS and CHI genes, whilst half carry AtPAL (3382, 5394, 5395) and the other half FjTAL (3968, 6455, 6456). Pathways were cloned into plasmid vectors with different antibiotic resistance markers. (**A**) Production of pinocembrin from pathways fed 3 mM phenylalanine. (**B**) Production of naringenin from pathways fed 3 mM tyrosine. Data represent the mean and standard deviation from four replicate cultures (wildtype DH5α cells grown at 30 °C for 24 hr in TBP media with 0.4% glycerol and 20 µg/ml cerulenin).

**Sup Figure 4**: Production of (2*S*)-flavanone targets from *trans*-phenylacrylic acid substrates (**A**) *trans*-phenylacrylic acids are converted to (2*S*)-flavanones through 3 sequential enzyme transformations. Six plasmid constructs were screened for production of (2*S*)-flavanones, differing only in the species origin of their 4CL genes. (**B**) Screening constructs for the conversion of cinnamic acid to pinocembrin. (**C**) Screening constructs for the conversion of coumaric acid to naringenin. (**D**) Screening constructs for the conversion of caffeic acid to eriodictyol. (**E**) Screening constructs for the conversion of ferulic acid to homoeriodictyol. (**F**) Screening constructs for the conversion of 4-methoxycinnamic acid to homoeriodictyol. Data represent the mean and standard deviation from four replicate cultures (wildtype MG1655 cells grown at 30 °C for 24 hr in TBP media with 0.4% glycerol, 20 μg/ml cerulenin and 3 mM *trans*-phenylacrylic acid substrate).

**Sup Figure 5**: Full combinatorial library of ferulic acid pathways built and tested in this study. Vector parts and plasmid numbers are given.

**Sup Figure 6**: *E. coli* production of homoeriodictyol and side-products. Constructs containing six different 4CL candidates were screened with the best-performing ferulic acid pathway (10692). (**A**) Final media titers of coumaric acid. (**B**) Final media titers of naringenin. (**C**) Final media titers of ferulic acid. (**D**) Final media titers of homoeriodictyol. Data represent the mean and standard deviation from four replicate cultures (wildtype DH5α cells grown at 30 °C for 24 hr in TBP media with 0.4% glycerol and 20 μg/ml cerulenin, in the presence or absence of 3 mM tyrosine).

**Sup Figure 7**: Schematic highlighting key target enzymes to enhance *E. coli* production of tyrosine. Genes highlighted in green were overexpressed from plasmid or genome-integrated constructs (* mutated to remove feedback inhibition). Genes in red were deleted from the DH5α host strain to increase flux toward tyrosine.

**Sup Table 1:** Kinetics information for TAL enzyme candidates. Known parameters for TAL, PAL and TAM reactions are shown.

**Sup Table 2**: List of genes used in this study.**Sup Table 3**: List of plasmids used in this study. Backbone, pathway components and origin of replications are given. For all genes the *in silico* Salis lab server was used to generate RBS sites and were fixed at 15000 RBS units for all RBS sites.

**Sup Table 3**: List of plasmids used in this study (continued).

**Sup Table 4**: List of strains used in this study.

**Sup Table 5**: MRM transitions and MS/MS operating parameters for LC–MS/MS analysis of targeted flavonoids and intermediates.


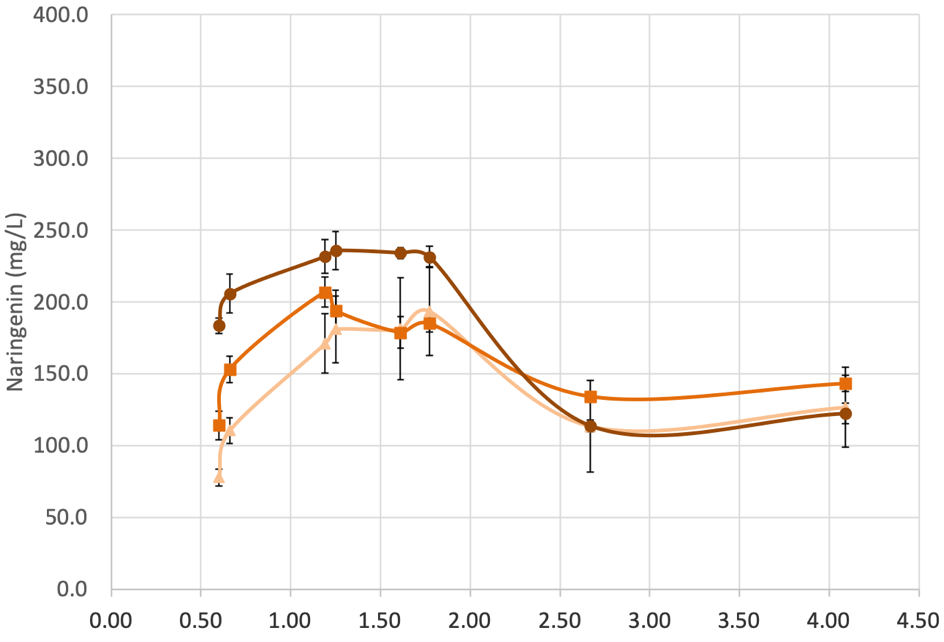

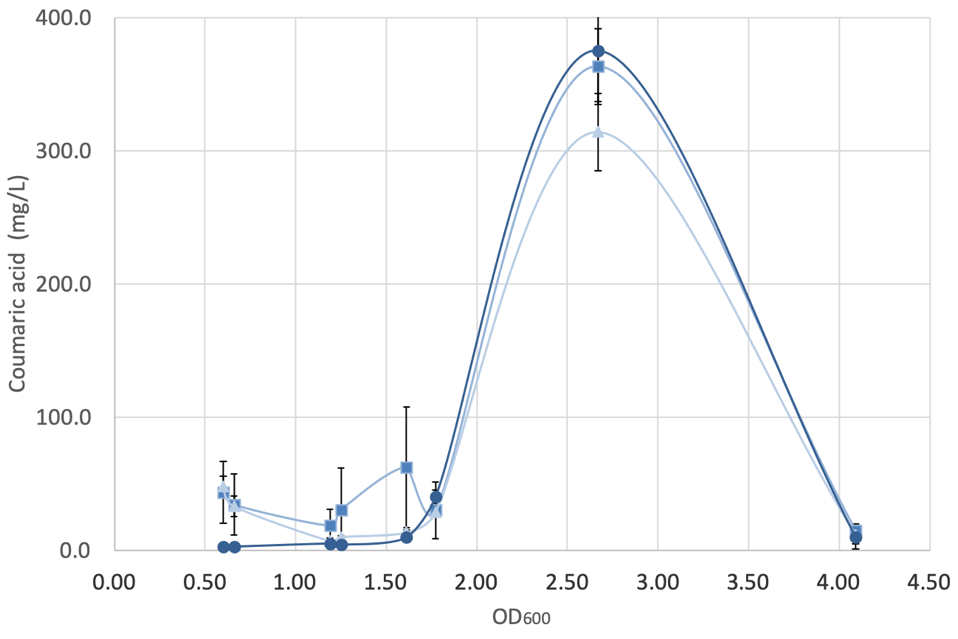


**Sup Figure 8**: Graph highlighting production titres at varies induction times for both Naringenin (top) and Coumaric acid (bottom). Circles, pathway 6456; Squares, pathway 6455; triangles, pathway 3968. Data represent the mean and standard deviation from three replicate cultures. All samples were harvest 24h post induction.
